# Clarifying Optimal Sodium InTake In Cardiovasular and Kidney (COSTICK) Diseases: a study protocol for two randomised controlled trials

**DOI:** 10.12688/hrbopenres.13210.1

**Published:** 2021-02-01

**Authors:** Andrew Smyth, Salim Yusuf, Claire Kerins, Colette Corcoran, Roisin Dineen, Alberto Alvarez-Iglesias, John Ferguson, Suzanne McDermott, Orlaith Hernon, Ritika Ranjan, Aoife Nolan, Matthew Griffin, Paula O'Shea, Michelle Canavan, Martin O'Donnell

**Affiliations:** 1 HRB Clinical Research Facility Galway, National University of Ireland, Galway, Galway, Ireland; 2 Population Health Research Institute, McMaster University and Hamilton Health Sciences, Hamilton, ON, Canada; 3 Department of Clinical Biochemistry, University Hospital Galway, Galway, Ireland

**Keywords:** Cardiovascular Disease, Chronic Kidney Disease, Sodium Reduction, Renal Insufficiency, Biomarkers, Clinical Trial

## Abstract

**Background:** While low sodium intake (<2.3g/day) is recommended for all, there is uncertainty about feasibility and net cardiovascular effects. In COSTICK, we evaluated the effects of a dietary counselling intervention (reduced sodium intake) on intermediate cardiorenal outcomes in patients with (STICK) and without (COSIP) mild/moderate kidney disease.

**Methods:** This is a protocol for two phase IIb randomised, two-group, parallel, open-label, controlled, single centre trials. Participants were aged >40 years with stable blood pressure, unchanged anti-hypertensive medications, willing to modify diet and provided written informed consent. Participants were excluded for abnormal sodium handling, heart failure, high dose diuretics, immunosuppression, pregnancy/lactation, postural hypotension, cognitive impairment, high or low body mass index (BMI) or inclusion in another trial. STICK participants had estimated glomerular filtration rate (eGFR) 30-60ml/min/1.73m ^2^ and were excluded for acute kidney Injury, rapidly declining eGFR; known glomerular disease or current use of non-steroidal anti-inflammatory drugs. For COSIP, participants were excluded for known kidney or cardiovascular disease. Participants were randomized to usual care only (healthy eating) or an additional sodium lowering intervention (target <100mmol/day) through specific counseling (sodium use in foods, fresh over processed foods, sodium content of foods and eating outside of home). In STICK the primary outcome is change in 24-hour urinary creatinine clearance. In COSIP, the primary outcome is change in five biomarkers (renin, aldosterone, high sensitivity troponin T, pro-B-type natriuretic peptide and C-reactive protein). Our primary report (COSTICK), reports six biomarker outcome measures in the entire population at 2 years follow-up.

**Discussion:** These Phase II trials will explore uncertainty about low sodium intake and cardiovascular and kidney biomarkers, and help determine the feasibility of low sodium intake. Trial results will also provide preliminary information to guide a future definitive clinical trial, if indicated.

**Trial registration: **STICK: ClinicalTrials.gov NCT02738736 (04/04/2016); COSIP: ClinicalTrials.gov NCT02458248 (15/05/2016)

## Introduction

Effective, simple, inexpensive interventions are essential for population-based strategies to impact blood pressure (BP), which may impact the global burden of cardiovascular disease (CVD) and chronic kidney disease (CKD) ^1^. Low sodium intake (<2.3g/day) is recommended for adults ^2^, with specific emphasis on individuals with hypertension, CVD and CKD. In populations with hypertension (or pre-hypertension) the evidence supporting low sodium intake (compared to moderate intake, 2.3–4.6g/day) is based on Phase II trials reporting a reduction in BP ^3– 6^.

Meta-analyses of observational studies estimate a mean BP reduction of 10/5mmHg per 2.3g (100mmol) reduction in daily sodium intake ^7^ and meta-analysis of clinical trials estimate that reducing from moderate to low sodium intake reduced BP by 5.0/2.7mmHg ^8^. It is assumed that all BP reductions will translate into reductions in CVD and CKD progression. However, there is also evidence of activation of the renin-angiotensin-aldosterone system (RAAS) ^9^ with low sodium intake from short-term studies (mostly <6 months), and prospective cohort studies report a J-shaped association of sodium intake with cardiovascular health ^1, 10, 11^. There have also been inconsistent reports about the association between sodium intake and biomarkers, including inflammatory biomarkers (C-reactive protein (CRP) and interleukin 6 (IL-6)), markers of cardiac injury/strain (high sensitivity troponin T (hsTnT) and pro B-type natriuretic peptide (proBNP) and of salt sensitivity (uromodulin) ^12– 15^.

For patients with CKD, no moderate or large-scale clinical trials have evaluated the effect of sustained low sodium intake on renal outcomes. Our systematic review of the association between dietary sodium intake/excretion and renal function showed that there is evidence of an association between high sodium intake, but not moderate intake, and glomerular filtration rate (GFR) decline among participants with CKD. Subsequent, analyses of additional observational studies reported no association between urinary sodium excretion and renal outcomes ^16– 18^. A further meta-analysis of clinical trials of dietary sodium reduction in CKD included 11 trials (n=738) and reported that moderate sodium restriction reduced blood pressure and urinary protein excretion ^19^.

These competing observations have cast considerable uncertainty about the optimal level of sodium intake, reflected in differences in guidelines and recommendations ^20, 21^. Therefore, the sustained effects of long-term low-sodium intake on biomarkers of cardiovascular and kidney function is a critical next step, necessary to inform the conduct of future definitive randomized controlled trials. 

## Protocol

### Study aim and design

Both trials were designed to explore the effects of an educational intervention to reduce sodium intake targeting low levels, compared to usual care. The Sodium In Take In Chronic Kidney Disease (STICK) clinical trial was designed to explore the effect of the intervention in adults with non-severe CKD on decline in renal function over two years. The Clarifying Optimal Sodium Intake in Populations (COSIP) clinical trial was designed to explore the effect of the intervention in adults without cardiovascular disease on a panel of cardiovascular biomarkers, over two years.

Both trials are Phase IIb randomised, two-group, parallel, open-label, controlled trials conducted at a single-centre. Blinding of participants and the research dietitian was not possible for practical reasons. Participants were recruited from hospital-based outpatient clinics or attending general practitioners in the catchment area of a tertiary referral hospital in Galway, Ireland. Potentially eligible participants were invited to participate and provided written informed consent before entering a screening period, consisting of an abbreviated food frequency questionnaire (FFQ; see extended data ^22^), medical history, laboratory investigations, baseline physical measurements (height, weight, body mass index (BMI), hip and waist circumference) and blood pressure (BP). Office BP was measured using a calibrated, automated oscillometric device and followed by a 24-hour ambulatory blood pressure monitor (ABPM) using the Spacelabs ABP 90217 device, where BP was measured every 30 minutes between 7am and 10pm, and every 60 minutes between 10pm and 7am. Given the complementary nature of both trials, participants were screened for both trials simultaneously, but only deemed eligible and randomized into STICK or COSIP. Rescreening of participants who failed the screening visit due to a blood pressure result or medication change was permitted after a minimum of three months.

### Eligibility criteria for both trials

Inclusion criteria for both trials included age >40 years, stable blood pressure, unchanged anti-hypertensive medications, willingness to modify dietary intake and written informed consent ( Table 1). Initially, we also required dietary assessed sodium intake of >2.3g/day, but removed this after the Vanguard phase due to weak correlation between the dietary sodium assessment and baseline 24 hour urine sodium collection. Exclusion criteria for both trials included abnormal sodium handling, heart failure, high dose diuretic use, immunosuppressive medication use, unable to comply with intervention or study visits, pregnancy or lactation, postural hypotension, cognitive impairment, high or low BMI or inclusion in another clinical trial.

**Table 1.** Eligibility Criteria.

	STICK	COSIP
**Inclusion Criteria**		
Age ≥40 years	✓	✓
Average Office BP<160/95mmHg	✓	✓
24hour average from ABPM<150/90mmHg	✓	✓
Anti-hypertensive and/or diuretic medications unchanged for three months	✓	✓
Self-reported willingness to modify dietary intake over sustained period	✓	✓
Written informed consent	✓	✓
Sodium Intake>2.3g/day from FFQ **	✓	✓
Most recent eGFR within 3 months	✓	✗
eGFR 30–60/min/1.73m ^2^ on ≥2 occasions ≥3 months apart	✓	✗
**Exclusion Criteria**		
Abnormal Sodium Handling (Bartter syndrome, syndrome of inappropriate antidiuretic hormone (SIADH) secretion, diabetes insipidus or hyponatraemia (serum sodium <125mmol/L)	✓	✓
Heart Failure (New York Heart Association (NYHA) Class III or IV symptoms or known left ventricular ejection fraction 30%)	✓	✓
Total daily diuretic therapy dose exceeding frusemide 80mg, bumetanide 2mg, hydrochlorothiazide 50mg, bendroflumethiazide 2.5mg, indapamide 2.5mg, metolazone 2.5mg or the use of both a loop and thiazide diuretic)	✓	✓
Use (within one month) of tacrolimus, cyclosporine, azathioprine or mycophenolate mofetil	✓	✓
Unable to follow educational advice	✓	✓
Prescribed high-salt diet, low-salt diet or sodium bicarbonate	✓	✓
Pregnancy or lactation	✓	✓
Unlikely to comply with study procedures or follow-up visits	✓	✓
Symptomatic or treated postural hypotension	✓	✓
Known dementia or cognitive impairment	✓	✓
BMI<20 or >40kg/m ^2^	✓	✓
Participating in another clinical trial or previous study allocation	✓	✓
Acute Kidney Injury (AKI) (doubling of baseline serum creatinine within 3 months) or Rapidly declining eGFR (≥10ml/ min/1.73m ^2^) over 6 months	✓	✗
Known Kidney Disease (Post-infectious glomerulonephritis, IgA nephropathy, thin basement membrane disease, Henoch-Schonlein purpura, proliferative glomerulonephritis, membranous nephropathy (including lupus), rapidly progressive glomerulonephritis, minimal change disease or focal segmental glomerulosclerosis) or dialysis or renal transplant (prior, current or planned))	✓	✗
NSAID use for >=3 days per week (excluding aspirin <100mg/day)	✓	✗
Known CKD or most recent eGFR≤60/min/1.73m ^2^	✗	✓
Previous Cardiovascular Disease (Myocardial infarction, percutaneous coronary intervention, coronary artery bypass grafting or stroke)	✗	✓

BP=Blood Pressure; ABPM=Ambulatory Blood Pressure Monitor; FFQ=Food Frequency Questionnaire; eGFR=estimated Glomerular Filtration Rate; BMI=Body Mass Index; NSAID=non-steroidal anti-inflammatory drug; CKD=chronic kidney disease **Sodium Intake removed from Inclusion Criteria during trial due to weak correlation between dietary sodium assessment and baseline 24hour urine sodium collection

### Eligibility criteria – STICK specific

All STICK participants were required to have a stable estimated glomerular filtration rate (eGFR) of 30–60ml/min/1.73m ^2^ within three months of randomization. Participants were excluded for recent acute kidney Injury (AKI), rapidly declining eGFR ^23^, known glomerular disease or current use of non-steroidal anti-inflammatory drugs (NSAID).

### Eligibility criteria – COSIP specific

For COSIP, participants were excluded for known CKD or CVD.

### Randomisation

Once confirmed eligible, participant details were entered into a trial-specific, secure, web-based randomisation service. In STICK, we used a minimization strategy (including previous attendance at a dietitian, diabetes, hypertension and strata of eGFR [30–39, 40–49 and 50–60ml/min/1.73m ^2^]) to support computer-generated block randomization with 1:1 assignment to intervention or usual care. In COSIP, we used computer-generated block randomization to 1:1 assign participants to intervention or usual care. Variable block sizes were used to minimize the occurrence of chance imbalances and to preserve allocation concealment. Eligible participants may have been randomised prior to attending the randomisation visit to facilitate the scheduling of separate intervention and usual care clinics.

### Study interventions

Usual care: All participants received a one-to-one, dietitian-developed healthy eating guidance session ^24^, administered by a trained member of the research team, over 15 minutes following randomization, in addition to written materials emphasizing key messages ^25^. Telephone contact was made at months nine and 15 to follow-up on key points on healthy eating (each 15 minutes) with a total contact time of 45 minutes over the course of the trials. Participants randomised to usual care did not meet with the dietitian nor did they receive focused recommendations on sodium intake (other than general guidance contained in 'Health Food for Life' ^25^).

Sodium reduction intervention: In addition to healthy eating guidance sessions, those randomised to the intervention received specific counseling on behavioural and environmental factors to promote reduction in sodium intake to a target of <100mmol/day (<2.3g/day). A research dietitian developed the specific components of the intervention, based on the trial of non-pharmacologic interventions in the elderly (TONE) trial ^26^. A registered dietitian delivered the intervention using an operations manual to standardize delivery within and across participants. In circumstances where the research dietitian was unavailable, an appropriately trained delegate delivered the intervention. The intervention targeted: (i) reducing use of ‘salt’ during food preparation (encouraging the use of herbs and/or spices); (ii) reducing table ‘salt’ use; (iii) encouraging fresh food consumption over processed or canned foods; (iv) identification of sodium content in foods; (v) modifying the consumption of foods with high sodium content; and (vi) advice on eating outside of home. After determining health literacy, using the Newest Vital Sign UK (NVS-UK) ^27^, participants were counselled and behaviour change techniques (goal setting, self-monitoring, frequent contact, feedback and re-enforcement, problem solving and motivational interviewing) were employed to reduce sodium intake by setting specific, measurable, achievable, realistic and timely (SMART) goals for the individual. Tools used included 24-hour dietary recall (analysed using Nutritics ^28^), three-day food diaries and sodium behavior checklists.

The intervention was delivered at all patient contacts up to and including T _7_ (21 month) visit ( Table 2) with approximately 225–255 minutes of in-person contact and 155 minutes of telephone contact over the course of the trials. Participants had in-person, one-to-one sessions with the study dietitian at randomization (T _0_), vanguard (T _V_), where relevant, and T _2_; group sessions at T _1 _and T _4_. In addition, each participant received five separate 15 min telephone calls (Week 1, Week 2, Week 4, Week 6 and Week 10) and 20 min telephone calls at T _3_, T _5_, T _6_ and T _7_.

**Table 2.** Usual Care and Intervention Sessions.

	Mode	Structure	Duration	Purpose
**Usual Care**				
T _0_ (Randomisation)	In Person	1:1	15 min	Provide Healthy Food for Life booklet and Food Pyramid Poster, set key messages
T _3_ (9 Months)	Phone call	1:1	15 min	Review key messages review progress/changes, troubleshoot
T _5_ (15 Months)	Phone call	1:1	15 min	Review key messages review progress/changes, troubleshoot
**Intervention**				
T _0_ (Randomisation)	In Person	1:1	60 min	Assess diet history, feedback, set SMART goals, fact sheet on salt, food label card
Call 1 (Week 1)	Phone call	1:1	15 min	Review SMART goals, problem solving, review goals
Call 2 (Week 2)	Phone call	1:1	15 min	Review SMART goals, review salt fact sheet, problem solving, review goals
Call 3 (Week 4)	Phone call	1:1	15 min	Review SMART goals, review salt fact sheet, check understanding of high sodium foods, problem solving, review goals
T _V_ (Vanguard) *	In Person	1:1	30 min	Review change from baseline, feedback, review SMART goals
Call 4 (Week 6)	Phone call	1:1	15 min	Review SMART goals, problem solving, review goals
Call 5 (Week 10)	Phone call	1:1	15 min	Review SMART goals, problem solving, review goals
T _1_ (3 Months)	In Person	Group	60 min	Presentation on reducing salt intake, case study discussion, participants identify key take home messages
T _2_ (6 Months)	In Person	1:1	60 min	Review change from baseline (+/- Vanguard), feedback, review SMART goals
T _3_ (9 Months)	Phone Call	1:1	20 min	Review content from T _1_, review SMART goals, review sodium habits, problem solving, review goals
T _4_ (12 Months)	In Person	Group	60 min	Presentation on reading food labels and identifying sodium content in foods, review own food packaging, review food label card
T _5_ (15 Months)	Phone Call	1:1	20 min	Review content from T _4_, review SMART goals, review sodium habits, problem solving, review goals
T _6_ (18 Months)	Phone Call	1:1	20 min	Review SMART goals, review sodium habits, problem solving, review goals
T _7_ (21 Months)	Phone Call	1:1	20 min	Review progress with SMART goals, review sodium habits, problem solving, review goals

* Vanguard visit applicable only for the first 20 participants

### Follow-up schedule

To minimize participant inconvenience and burden, six or seven in-person visits were considered essential (screening, randomization (T _0_), T _v_ (first 20 participants for each trial), T _1_, T _2_, T _4_ and T _8_) and the remaining four follow-up visits were carried out by telephone (T _3_, T _5_, T _6_ and T _7_) ( Table 3). Post-trial, participants will return to routine clinical care. 

**Table 3.** Schedule of Events.

	T _-1_	T _0_	**T _V_	T _1_	T _2_	T _3_	T _4_	T _5_	T _6_	T _7_	T _8_
	Screening	Time 0	1 Month	3 Months	6 Months	9 Months	12 Months	15 Months	18 Months	21 Months	24 Months
Eligibility Criteria	X										
Informed Consent	X										
Demographics	X	X									X
Medications	X	X	X	X	X	X	X	X	X	X	X
Physical Measurements	X						X				X
Office BP	X		X	X	X		X				X
Abbreviated FFQ	X										X
24-hour ABPM	X										X
eGFR	X	X	X	X			X *				X
Healthy Eating Advice		X				X		X			
Eligibility Check		X									
Randomisation		X									
Medical History		X									
Blood Biochemistry		X	X	X							X
Proteinuria		X					X				X
Biobank Samples		X		X							X
24-hour urine		X	X	X	X		X				X
2 ^nd^ morning fasting urine		X					X				X
In person visits	X	X	X	X	X		X				X
Telephone visit						X		X	X	X	
Adherence and Outcomes			X	X	X	X	X	X	X	X	X

BP=Blood Pressure; FFQ=Food Frequency Questionnaire; ABPM=Ambulatory Blood Pressure Monitor; eGFR=estimated Glomerular Filtration Rate) *STICK participants only; **Vanguard study visit applicable only for the first 20 participants

### Data collection

The following data were collected using standardised case report forms (CRF): demographics, physical measurements (BP, height, weight, waist and hip circumference), medications, medical history, lifestyle factors, randomization allocation, diet, delivery of healthy eating guidance session, delivery of the sodium reduction intervention, clinical events, hospitalisations, adverse events and laboratory results (see extended data ^22^). Diet was measured using FFQ, food diaries and a questionnaire focused on sodium-specific behaviours (see extended data ^22^). Laboratory samples were analysed at the Galway University Hospital laboratory using standardised storage, handling and analytical procedures. We measured serum sodium, potassium, creatinine, glycated haemoglobin (HbA1c), high-density lipoprotein (HDL), low-density lipoprotein (LDL), plasma renin activity, aldosterone, high sensitivity troponin T (hsTnT), Pro B-type Natriuretic Peptide (proBNP), C-reactive protein (CRP), urine protein creatinine ratio (uPCR), 24 hour urine (sodium, potassium, creatinine and protein) and fasting morning urine (sodium, creatinine and protein). This included serum creatinine, measured using methodology traceable to an isotope dilution mass spectrometry (IDMS) reference measurement. Samples were transported to the laboratory within one hour of blood draw and analyses were conducted using standardized laboratory methods on the Roche Cobas® 8000 modular analyser series (Roche Diagnostics Limited, West Sussex, UK), except for HbA1c which was measured using capillary electrophoresis on the Sebia Capillarys 3 automated haemoglobin analyser using the Sebia HbA1c kit (2017/05). After additional consent was obtained, additional blood and urine samples for PAXgene samples for RNA measurement and for biobanking were taken at T _0_, T _1_ and T _8_. To ensure high quality data collection, staff received training on completing paper-based CRF and additional training on entering data into the study database, which included database integrity checks to ensure data consistency.

### Study outcomes

In STICK, the primary outcome measure is change in 24-hour urinary creatinine clearance over two years. We adopt this approach, rather than eGFR, to improve reliability and validity of the primary outcome measure. In COSIP, the primary outcome measure is change in cardiovascular biomarkers (renin, aldosterone, hsTnT, proBNP and CRP) over two years. For COSTICK, the primary outcome measures are creatinine clearance, renin, aldosterone, hsTnT, proBNP and CRP. Secondary outcomes included change in (i) 24 hour ABPM, (ii) 24 hour urine sodium excretion, (iii) 24 hour urine protein, (iv) Modification of Diet in Renal Disease (MDRD) eGFR ^23^, (v) Chronic Kidney Disease Epidemiology Collaboration (CKD-EPI) eGFR ^29^, (vi) Kidney Disease Improving Global Outcomes (KDIGO) CKD risk category ^30^ and (vii) change in PAXgene RNA, and number of (viii) cardiovascular events (acute coronary syndrome, myocardial infarction, heart failure or stroke), (ix) syncope events (prescyncope, syncope, hypotension or orthostatic hypotension) and (x) renal events (acute kidney injury, dialysis, transplant or end stage renal disease).

### Safety reporting

An adverse event is defined as any untoward medical occurrence, including an exacerbation of a pre-existing condition or abnormal laboratory finding, with or without a causal relationship with sodium intake. Participants were monitored for adverse events from informed consent through completion of the final study visit. All adverse events were captured on case report forms, entered into the clinical trial database and coded using the Medical Dictionary for Regulated Activities (MedDRA). Annual line listings of adverse events were reported to the ethics committee.

### Withdrawal of intervention

The study intervention was withdrawn if: (i) symptomatic orthostatic hypotension with systolic BP ≤100mmHg; (ii) diagnosis of glomerular disease (as listed in exclusion criteria); (iii) diagnosis of ESRD, initiation of dialysis or renal transplantation; (iv) prescription of sodium bicarbonate therapy or treating physician recommended against sodium restriction; (v) pregnancy; or (vi) acquired new dietary requirement that precluded the intervention. These participants were encouraged to complete all remaining follow-up visits.

### Sample size calculation

For STICK, we assumed a mean decline in creatinine clearance of 8±5ml/min/1.73m ^2^ over the trial period ^31^ and estimated a minimum clinically meaningful effect size of 25% relative reduction. Based on an alpha of 0.05 and power of 80%, a per group sample size of 99 participants was required. Assuming a dropout rate of 5% participants, a net crossover/non-adherence of 5% in favour of the control group, we required a total sample size of 224 participants. For COSIP, we based sample size on the ability to detect an effect size of 0.40 in the between-group difference in mean change scores of biomarkers (80% power and alpha 0.05), equating to a difference of 0.4 of the standard deviation of the change in biomarker. As the COSIP trial is considered exploratory, each biomarker is tested at significance threshold p<0.05. Assuming a dropout rate of 5% participants, a net crossover/non-adherence of 15%, we require a total sample size of 286 participants. Combining COSIP and STICK trials resulted in the ability to detect a smaller effect size.

### Statistical analysis plan

Descriptive statistics will be used to describe baseline characteristics, the flow of trial participants and the amount of missing data for predictor and outcome variables. All losses to follow-up and dropouts will be accounted for and reasons documented. Participants randomised but who did not attend the randomisation visit will be included in analyses. Suitable transformations will be used if the underlying normality assumption is a concern and the bootstrap procedure and a comparison of the median change will be used if a suitable transformation cannot be made. All tests of significance will be two-sided using an alpha of 0.05 for statistical significance and will not be adjusted for multiple testing due to the exploratory nature of the trials. The primary analysis will use the intention-to-treat dataset for all outcomes. A secondary per-protocol analysis will also be carried out, with post randomisation exclusions due to ineligibility, non-compliance, loss to follow-up or missing data.

The primary analysis will consist of fitting a separate linear, multivariable analysis to each of the six outcomes: renin, aldosterone, creatinine clearance, pro-BNP, troponin, and CRP, and secondary outcome measure of 24-hour ABPM. We will review data for implausible values and exclude extreme changes (e.g. change in renin or aldosterone impacted by change in medications). Corresponding baseline measurements of each biomarker will be included as covariates in the model, along with a factor variable for the sub-study in which each patient was recruited, age, sex and whether the participant had attended a dietitian in the 6 months previous to baseline (incident vs prevalent participant). To ensure estimates are more robust to potential effects of missing data at 24-month follow-up, an inverse probability weighting will be applied in all regression analyses to account for the differing propensity of patients with certain baseline characteristics to be included in the final analysis. All covariates included in the regression model will be included in the missingness model, including the other 5 primary endpoints. 

Reporting of primary analysis results will consist of tabulations of the unadjusted and adjusted mean differences at 24 months between intervention and control groups for each biomarker, with confidence intervals of these parameter estimates and p-values, along with descriptive unadjusted mean differences at 24 months between intervention and control groups. A forest plot of the standardised, adjusted mean difference for all 6 endpoints will also be generated. Statistical tests of interaction (Wald) will be used for all subgroup analyses. A sensitivity analysis will be performed to assess the effect of participant reported compliance on treatment effect. Due to the duration of this trial, we expect to have a complete dataset for covariate and outcome data for 90% of participants. Complete case analysis will be used to handle missing data. A sensitivity analysis with multiple imputation will also be carried out if there is >10% missing data for covariates.

### Data monitoring

A data and safety monitoring board (DSMB), chaired by a clinician scientist with experience in clinical trials, reviewed the progress of the trials and safety data when 50% of participants completed one-year follow-up, when the DSMB recommended that the trials proceed. An independent study monitor reviewed the integrity of study data at multiple timepoints throughout the trials and ensured that study procedures were compliant with the International Conference on Harmonisation Good Clinical Practice (ICH-GCP).

### Study administration

These trials are single centre studies with all activities conducted and coordinated at the HRB Clinical Research Facility Galway (HRB-CRFG). The investigators did not implement any deviation from, or changes to, the trial protocols without review and documented approval from the research ethics committee. All participants provided written informed consent before any study specific activities were completed, and all data was handled with strict privacy and electronic data security standards, using unique subject identifiers to prevent unauthorised identification of research participants. Pseudononymised data were entered into a study-specific, secure, password-protected clinical trial database with an audit trail.

### Dissemination of information

The results of the trials will be presented at scientific meetings (local, national and international), patient-oriented meetings and published in peer-reviewed publications. The HRB-CRFG and investigators have the ownership of all data and results collected during the trials and have full rights to publication, without restriction. For all publications and presentations from this work, authors will be required to make substantial contributions to (i) concept or design; acquisition, analysis or interpretation of data; (ii) drafting or critically revising manuscripts and (iii) give final approval of the version to be published, in line with the International Committee of Medical Journal Editors guidelines for authorship. The datasets will not be made publicly available, instead they will be maintained at HRB-CRFG and the investigators must review and approve all requests for access to the data.

### Study status

Recruitment of both clinical trials is closed and the final visits of all patients have taken place. Data cleaning is ongoing, ahead of database lock, statistical analyses and trial reporting.

## Discussion

Clinical trials that challenge current guidelines are difficult but are of considerable importance, with major implications for management of patients and populations. For those consuming high sodium diets (>200mmol/day), there is convincing evidence of negative associations including reduced eGFR ^32, 33^, increased proteinuria ^32, 34, 35^ and adverse associations with blood pressure and CVD. However, there is major uncertainty about whether low sodium intake is associated with more benefit than harm, which needs to be interrogated with clinical trials. Dietary trials over medium to long durations are particularly challenging, but data from rigorously conducted trials are necessary to appropriately inform guidelines. These trials will help determine the feasibility of sodium restriction to current guideline levels and determine if this reduction in intake is sustainable. The results will provide preliminary information, to guide future definitive clinical trials, if indicated.

Dietary sodium intake is modifiable, as evidenced by numerous clinical trials of standardized educational interventions that successfully reduced dietary sodium intake to low intake range over the short-term ^26, 36^, including a small, short duration feasibility clinical trial in CKD ^37^. It is clear from such trials that a sodium reduction intervention strategy will reduce sodium intake, but the amount and sustainability of the reduction is less clear. We designed our intervention based on the TONE trial ^26^, targeting non-discretionary sources of sodium intake. After the Vanguard phase (first 20 participants in each trial) was completed, we observed that mean 24 hour urine sodium excretion reduced from 163(62)mmol/day to 125(53)mmol/day, suggesting that the intervention was effective in lowering sodium intake. 

Our trials have a number of limitations. First, the trials are open-label as it wasn’t practical to blind participants, investigators or study staff to the intervention. However, to minimize the risk of bias we will perform blinded outcome assessment including blinding of the trial statistician. Second, participants in the usual care arm may receive non-study recommendations on sodium reduction, reducing the likelihood of observing the effect from our intervention, biasing toward the null. To minimise this risk of bias, treating clinicians were asked not to explicitly discuss sodium reduction with participants unless strongly indicated and the trial intervention was to be withdrawn. Similarly, as contamination is a threat to trial validity, only participants randomized to the intervention met with the study dietitian, participants were instructed not to discuss the educational sessions with other participants and participants were invited to attend follow-up visits on days based on study allocation. Third, there is a risk of volunteer bias, as participants may differ from non-participants, impacting trial generalizability. We will measure the potential effect of this bias by comparing non-participants and participants. Fourth, the primary outcome measures are changes in biomarkers rather than hard clinical endpoints, which would require a trial of longer duration (to accumulate a sufficient number of clinical events) to demonstrate a treatment effect. The findings of our trials can be used to inform future, larger, multicenter trials. Fifth, individuals with CKD may have previously consulted with a dietitian and received advice on lowering sodium intake, limiting their ‘responsiveness’ to our intervention. Therefore, we included previous dietitian review in the minimization strategy for randomization in STICK; in addition, we believe that the trial intervention is more intensive than standard care. Finally, there is a risk of non-compliance with the intervention and a reliance on participant self-reported adherence as this is a behavioural intervention trial. To maximise compliance, we: (i) self-selected participants most likely to adhere to a behavioural intervention; (ii) implemented standardised telephone follow-up to provide additional advice and support, answer questions, reinforce key messages and remind participants about visits; and (iii) provided written materials on the study interventions. We also completed multiple 24-hour urine collections for sodium to measure objectively measure adherence, although these collections also have limitations ^38^.

*Ethics approval and consent to participate:* The STICK trial was conducted in accordance with (ICH-GCP) ^39^ and the trial protocol received approval from the Galway University Hospitals Research Ethics Committee (Ref C.A. 1267). The COSIP trial was conducted in accordance with (ICH-GCP) ^39^ and the trial protocol received approval from the Galway University Hospitals Research Ethics Committee (Ref C.A. 1182). Written informed consent was obtained from all participants before any study-specific activity was carried out.

## Data availability

### Underlying data

No data are associated with article

### Extended data

Open Science Framework: Clarifying Optimal Sodium InTake In Cardiovasular and Kidney (COSTICK). https://doi.org/10.17605/OSF.IO/HYKT5 ^22^

This project contains the following extended data:

- 24 Month Sodium Checklist with barcode V2.0 190117.pdf (Sodium Behaviour Checklists – 24 month)
- All visit CRFs combined.pdf (Trial Case Report Forms)
- FFQ.pdf (Food Frequency Questionnaire)
- Randomisation Sodium Checklist with barcode V2.0 190117.pdf (Sodium Behaviour Checklists - Randomisation)

### Reporting guidelines

Open Science Framework: SPIRIT Checklist for “ Clarifying Optimal Sodium In Take In Cardiovasular and Kidney (COSTICK) Diseases: a study protocol for two randomised controlled trials”, https://doi.org/10.17605/OSF.IO/HYKT5 ^22^

Data are available under the terms of the Creative Commons Zero “No rights reserved” data waiver (CC0 1.0 Public domain deciation).
