# Supplementary material for: Clarifying Optimal Sodium InTake In Cardiovasular and Kidney (COSTICK) Diseases: a study protocol for two randomised controlled trials
Source: HRB Open Res. 2022 Feb 7;4:14. Originally published 2021 Feb 1. [Version 2] doi: 10.12688/hrbopenres.13210.2 (PMC9623192; doi:10.12688/hrbopenres.13210.2)
Supplement: Peer review history

# Peer review: Reviewer response for version 2

**DOI:** 10.21956/hrbopenres.14728.r32986

**Published:** 2022-10-31

**Authors:** Javier Mariani

**Affiliations:** 1 Hospital de Alta Complejidad en Red El Cruce - Néstor Kirchner, Florencio Varela, Argentina; 2 Hospital Británico de Buenos Aires, Buenos Aires, Argentina

The authors present the revised version of a protocol for two RCTs assessing an intervention to reduce sodium intake on renal and cardiovascular markers outcomes. The protocol is the joint analysis of two randomized clinical trials of dietary counselling among participants with chronic kidney disease (STICK) and primary prevention (COSIP). The study hypothesis is relevant and, as correctly pointed out by the authors, there is a need for evidence on the optimal level of sodium intake in these populations. The methods are exposed and allow for to reproduce the study. The manuscript is comprehensive and well written.

**Minor points:**

- It would be useful to present the subgroups that will be analyzed in the subgroup analysis.

- There is a parenthesis lacking in the exclusion criterion "Known Kidney Disease (Post-infectious glomerulonephritis...".

Is the study design appropriate for the research question?

Yes

Is the rationale for, and objectives of, the study clearly described?

Yes

Are sufficient details of the methods provided to allow replication by others?

Partly

Are the datasets clearly presented in a useable and accessible format?

Not applicable

Reviewer Expertise:

Cardiology. Epidemiology.

I confirm that I have read this submission and believe that I have an appropriate level of expertise to confirm that it is of an acceptable scientific standard.

# Peer review: Reviewer response for version 1

**DOI:** 10.21956/hrbopenres.14362.r31005

**Published:** 2021-12-20

**Authors:** Maristela Böhlke

**Affiliations:** 1 Post-Graduate Program in Health and Behavior, Catholic University of Pelotas, Pelotas, Brazil

Smyth *et al.* describe in this manuscript the research protocol for two randomized controlled trials designed to evaluate the effects of dietary counseling to reduce sodium intake on surrogate cardiorenal outcomes in patients with and without stage III chronic kidney disease. The study has been already concluded and the authors state that the data analysis has not yet been performed.   

 The research question is highly relevant considering the current state of the discussion about sodium intake. The upper limits for sodium intake advised for scientific societies have increased in their updated reports and diverge between them due to controversies in study results, but there also seems to be some economic pressure and deductive reasoning from anthropological data, alongside the best scientific findings.

    

**Introduction**

- The authors state ' *However, there is also evidence of activation of the renin-angiotensin aldosterone system (RAAS)* *with low sodium intake from short-term studies (mostly <6 months)'* but in fact, this RAAS activation is also well documented in situations of low sodium intake for a long time, as occurs among Yanomami tribes in northwestern Brazil.

**Methods**

 Eligibility Criteria

- Why was the use of azathioprine or mycophenolate use exclusion criteria? Should not the use of SGLT2 inhibitor be an exclusion criterion?
- What is the reference for the upper limit for the dose of diuretic to be considered high?
- The meaning of the statement ' *Eligible participants may have been randomised prior to attending the randomisation visit to facilitate the scheduling of separate intervention and usual care clinics' *is not clear.

Intervention

- Randomization should be to similar diets, with the only difference being the reduction in the sodium intake, as a low-sodium diet may differ in other dietary factors, such as potassium intake, which has also been associated with cardiorenal endpoints.

Data Collection

- There is not enough information about the collection and analysis of laboratory variables, especially for hormones and enzymes, which depend on body position and time of day to interpret the results.
- In the Data Collection section, there is the statement ' *After additional consent was obtained, additional blood and urine samples for PAXgene samples for RNA measurement and for biobanking were taken at T0, T1 and T8.'. *with no prior mention of RNA analysis, details on methodology or type of genetic analysis to be performed. Please clarify.

Study Outcomes

- As there is some evidence that sodium restriction also has adverse effects of activating the renin angiotensin aldosterone system, increasing catecholamines, and adversely affecting insulin sensitivity and lipids, the inclusion of some measure of insulin sensitivity as an outcome would be desirable.  

Sample Size Calculation

- It is not clearly explained, and the calculation report does not support the minimal number of participants that the authors describe. If the calculation has not been done prior to the randomization, a power calculation may be an acceptable option due to the exploratory nature of the trial.

Statistical Analysis Plan

- Considering that a low-sodium diet may have different effects according to the subgroup of individuals, I suggest an exploratory analysis of subgroups, especially for diabetic patients and patients using angiotensin converting enzyme inhibitors or angiotensin receptor blockers. There is evidence that a low-sodium diet is associated with increased cardiovascular and all-cause mortality in diabetic patients, due to greater activation of the sympathetic nervous system and RAAS, in addition to greater insulin resistance.

Is the study design appropriate for the research question?

Yes

Is the rationale for, and objectives of, the study clearly described?

Partly

Are sufficient details of the methods provided to allow replication by others?

Partly

Are the datasets clearly presented in a useable and accessible format?

Not applicable

Reviewer Expertise:

Nephrology

I confirm that I have read this submission and believe that I have an appropriate level of expertise to confirm that it is of an acceptable scientific standard, however I have significant reservations, as outlined above.

# Response

**Published:** 2022-02-03

**Authors:** Andrew Smyth

**Affiliations:** National University of Ireland, Galway, Ireland

**Comment: **The authors state ' *However, there is also evidence of activation of the renin-angiotensin aldosterone system (RAAS) with low sodium intake from short-term studies (mostly <6 months)'* but in fact, this RAAS activation is also well documented in situations of low sodium intake for a long time, as occurs among Yanomami tribes in northwestern Brazil.

**Response: **We thank the reviewer for this comment and we have provided additional information, including mention of the Yanomami tribes, to the introduction.

**Comment:** Why was the use of azathioprine or mycophenolate use exclusion criteria? Should not the use of SGLT2 inhibitor be an exclusion criterion?

**Response: **Azathioprine and mycophenolate mofetil were exclusion criteria because of potential impacts on urinary excretion of sodium. At the time of trial design and recruitment, which began in March 2016, SGLT2 inhibitors were not in widespread use. We agree with the reviewer that SGLT2 inhibitors would be an additional exclusion criterion if we were to design a further trial.

**Comment:** What is the reference for the upper limit for the dose of diuretic to be considered high?

**Response: **Details on the upper limit for dose of individual diuretics is provided in Table 1 Eligibility Criteria.

**Comment: **The meaning of the statement ' *Eligible participants may have been randomised prior to attending the randomisation visit to facilitate the scheduling of separate intervention and usual care clinics' *is not clear.

**Response: **We have restructured this sentence to improve clarity. Some participants were randomised in advance of the randomisation visit to facilitate clinic scheduling, as participants randomised to the low sodium intervention met with the study dietitian but those randomised to usual care did not.

**Comment: **Randomization should be to similar diets, with the only difference being the reduction in the sodium intake, as a low-sodium diet may differ in other dietary factors, such as potassium intake, which has also been associated with cardiorenal endpoints.

**Response: **All participants in the trial (low sodium and usual care) received dietary counselling to enhance the principles of healthy eating and to enforce key messages and national dietary guidelines. This was conducted to ensure that our participants had similar overall dietary advice. Our participants were randomised to receive specific counselling targeting sodium intake or not (usual care).

**Comment: **There is not enough information about the collection and analysis of laboratory variables, especially for hormones and enzymes, which depend on body position and time of day to interpret the results.

**Response: **We provided additional detail on the collection of blood samples and the protocol used

**Comment: **In the Data Collection section, there is the statement ' *After additional consent was obtained, additional blood and urine samples for PAXgene samples for RNA measurement and for biobanking were taken at T0, T1 and T8.'. *with no prior mention of RNA analysis, details on methodology or type of genetic analysis to be performed. Please clarify.

**Response: **These samples were obtained and stored for potential future use and are not part of the primary trial report.

**Comment: **As there is some evidence that sodium restriction also has adverse effects of activating the renin angiotensin aldosterone system, increasing catecholamines, and adversely affecting insulin sensitivity and lipids, the inclusion of some measure of insulin sensitivity as an outcome would be desirable.

**Response: **We designed the trials around the outcomes of cardiovascular and renal function. We did not complete measurements of catecholamines or insulin sensitivity during the trial. We thank the reviewer for raising these issues, which we will consider exploring in future posthoc work with the biobanked samples.

**Comment: **It is not clearly explained, and the calculation report does not support the minimal number of participants that the authors describe. If the calculation has not been done prior to the randomization, a power calculation may be an acceptable option due to the exploratory nature of the trial.

**Response: **The rationale and underlying assumptions (alpha, power, expected baseline change in biomarker, clinically meaningful effect size, dropout and potential for crossover/nonadherence) are provided for the STICK and COSIP trials.

**Comment: **Considering that a low-sodium diet may have different effects according to the subgroup of individuals, I suggest an exploratory analysis of subgroups, especially for diabetic patients and patients using angiotensin converting enzyme inhibitors or angiotensin receptor blockers. There is evidence that a low-sodium diet is associated with increased cardiovascular and all-cause mortality in diabetic patients, due to greater activation of the sympathetic nervous system and RAAS, in addition to greater insulin resistance.

**Response: **We thank the reviewer for this comment. The primary report of the trial results will include subgroup analyses and we will distinguish between pre-specified and post-hoc subgroup analyses.

# Peer review: Reviewer response for version 1

**DOI:** 10.21956/hrbopenres.14362.r28822

**Published:** 2021-02-22

**Authors:** Niels Graudal

**Affiliations:** 1 Copenhagen Lupus and Vasculitis Clinic, Copenhagen University Hospital, Copenhagen, Denmark

**Introduction**

 In this study protocol of two randomized controlled trials, which intend to investigate the optimal sodium intake in cardiovascular and kidney diseases, the authors correctly state that the evidence supporting low sodium intake (compared to moderate intake, 2.3–4.6g/day) is based on Phase II trials reporting a reduction in BP. However, it should be possible to cite references, which better support that statement, than those mentioned (references 3-6).

 The authors state that there is also evidence of activation of the renin-angiotensin-aldosterone system (RAAS) with low sodium intake from short-term studies (mostly <6 months). In this context it might be worth mentioning that a culture on life-long low sodium intake has a strong activation of RAAS, indicating that this activation is not just a short-term effect, but a persistent effect (reference: Circulation 1975; 52: 145-151).

 The authors also state that prospective cohort studies report a J-shaped association of sodium intake with cardiovascular health. One of the mentioned references does not support that view, as this analysis does not investigate the independent association of a low sodium intake with health outcomes, but only the association of a high sodium intake (reference 10).

 The authors give a reference to their systematic review of the association between dietary sodium intake/excretion and renal function. It would be helpful to mention this reference, when mentioned the first time in the introduction, i.e. move reference 32 to reference 16. In this context the authors may benefit from consulting two additional references: *Kidney International 2018; 93: 921-931* and *Kidney International 2018; 93: 776-778*, to further explore the known evidence of the association between sodium intake and renal function.

 Reference 20 is not a guideline or a recommendation, but a commentary, which modifies an Institute of Medicine report commissioned by the Centre of Disease Control and Prevention to review and assess potential benefits and adverse outcomes of reducing sodium intake in the population (Institute of Medicine. 2013. *Sodium Intake in Populations: Assessment of Evidence.* Washington, DC: The National Academies Press, 2013). Likewise, reference 21 is also an opinion paper. It would be more precise to mention the original IOM report and to mention that there is a discrepancy between the inconclusive outcome of the original commissioned independent report intended to form the basis for the guidelines and the final definitive conclusion of the guidelines (reference: Dietary Guidelines for the Americans

https://health.gov/dietaryguidelines/2015/guidelines/ (2015-2020).

**Study aim and outcome**

 The purpose was to explore the effects of an educational intervention to reduce sodium intake targeting low levels. This is the right question, because the recommendations are not just to lower sodium intake, but to lower sodium intake to low levels below 2.3 g, the problem being that there are no studies to support beneficial health effects of such a reduction. Furthermore, the COSTICK study includes an outcome, which is not just a “biochemical” outcome, but a true morbidity outcome, as it reflects the effect on the renal function over a 2-year period. In contrast, the COSIP study “only” reflects cardiovascular biomarkers, but some of them have not been invetigated previously and may contribute to establish whether a low sodium diet changes the disposition (for instance increases or decreases hsTnT and proBNP) to true cardiovascular events. Although it must be expected that aldosterone and renin will increase during the low sodium diet according to reference 9 and a new study (DOI: https://doi.org/10.1016/j.eclinm.2021.100750) ^1^, it is worth to investigate this effect over a 2-year period.

**Study intervention**

 The diet counselling is more intense in the intervention group, which leaves the question whether there will be other dietary differences between the two groups than differences in sodium intake (Table 2). Such interactions may complicate the final interpretation of the results. The many 24-h urine measurements (Table 3) eliminate one of the previous issues of conflict (the method of sodium intake measurement), which has resulted in reservations on the interpretation of outcomes in previous studies.

**Discussion**

 The study is well designed, the statistical procedures are appropriately described, and the level of sodium reduction is controlled by multiple 24-h urine measurements. These preconditions should ensure solid conclusions.

 The authors state that “These trials will help determine the feasibility of sodium restriction to current guideline levels and determine if this reduction in intake is sustainable.” This is an important point, supported by the fact that the duration of sodium reduction in RCTs sponsored by the National Heart, Lung and Blood Institute decreased from 36 months (The Hypertension Prevention Trial: *Arch Intern Med* 1990; 150: 153–162) to 6 months (The Trials of Hypertension Prevention, *JAMA* 1992; 267: 1213–1220 and *Arch Intern Med *1997; 157: 657–667) to 1-month (the Dietary Approaches to Stop Hypertension (DASH) diet *N Engl J Med* 2001; 344: 3–10). The obvious explanation for this change in duration strategy from 36 months to 1 month is that the sodium reduction and blood pressure effects achieved in the longer-term HPT and TOHP studies were small. This leaves some questions. Why is the official policy to reduce sodium intake to below 2.3 g, if this strategy is not feasible for more than 1 month, even under controlled circumstances? And why did NHLBI support a short-term sodium reduction study, knowing that a sodium reduction strategy to below 2.3 g was not feasible for a longer period? In this context it will be exciting to learn whether the present studies will show that such a strategy is possible or not, and it will be important to interpret the outcomes in relation to the achieved sodium reduction. If the goal to reduce sodium intake to below 2.3 g is not achieved, it is reasonable to raise the question why it is difficult to get below that level. Is it because the normal human neuro-endocrinological physiology counteracts a sodium intake below 2.3 g, simply because the net-effect of such a low sodium intake is harmful, as indicated by the activation of RAAS at sodium intakes below 2.3 g? In this context it is predictable that renin and aldosterone will only increase significantly, if the goal to reduce sodium intake below 2,3 is fulfilled.

 From a scientific and evidence-based point of view the outcomes of the COSTICK and COSIP studies will be exciting. If the studies show the “right” results and support the present sodium reduction policy, the studies will be well-received by the health authorities. But, if the results “wrongly” are in opposition to the existing sodium reduction policy, they will have no impact, because the political decision makers in the health organizations for decades have explained away studies, which do not confirm the official policy.

Is the study design appropriate for the research question?

Yes

Is the rationale for, and objectives of, the study clearly described?

Yes

Are sufficient details of the methods provided to allow replication by others?

Yes

Are the datasets clearly presented in a useable and accessible format?

Not applicable

Reviewer Expertise:

Health effects of sodium

I confirm that I have read this submission and believe that I have an appropriate level of expertise to confirm that it is of an acceptable scientific standard.

## References

[1] : Influence of sodium intake and change in sodium intake on plasma-renin in man. *EClinicalMedicine* .2021; 10.1016/j.eclinm.2021.100750 10.1016/j.eclinm.2021.100750 PMC802015533842863

# Response

**Published:** 2022-02-03

**Authors:** Andrew Smyth

**Affiliations:** National University of Ireland, Galway, Ireland

**Comment: **In this study protocol of two randomized controlled trials, which intend to investigate the optimal sodium intake in cardiovascular and kidney diseases, the authors correctly state that the evidence supporting low sodium intake (compared to moderate intake, 2.3–4.6g/day) is based on Phase II trials reporting a reduction in BP. However, it should be possible to cite references, which better support that statement, than those mentioned (references 3-6).

**Response: **We have updated our references in line with the recommendation of the reviewer.

**Comment: **The authors state that there is also evidence of activation of the renin-angiotensin-aldosterone system (RAAS) with low sodium intake from short-term studies (mostly <6 months). In this context it might be worth mentioning that a culture on life-long low sodium intake has a strong activation of RAAS, indicating that this activation is not just a short-term effect, but a persistent effect (reference: Circulation 1975; 52: 145-151).

**Response: **We added additional information to the sentences describing activation of RAAS and updated the references.

**Comment: **The authors also state that prospective cohort studies report a J-shaped association of sodium intake with cardiovascular health. One of the mentioned references does not support that view, as this analysis does not investigate the independent association of a low sodium intake with health outcomes, but only the association of a high sodium intake (reference 10).

**Response: **Reference 10 has been removed.

**Comment: **The authors give a reference to their systematic review of the association between dietary sodium intake/excretion and renal function. It would be helpful to mention this reference, when mentioned the first time in the introduction, i.e. move reference 32 to reference 16. In this context the authors may benefit from consulting two additional references: *Kidney International 2018; 93: 921-931* and *Kidney International 2018; 93: 776-778*, to further explore the known evidence of the association between sodium intake and renal function.

**Response: **We have altered our references to include our systematic review at an early stage and include the additional references on the association between sodium intake and renal function.

**Comment: **Reference 20 is not a guideline or a recommendation, but a commentary, which modifies an Institute of Medicine report commissioned by the Centre of Disease Control and Prevention to review and assess potential benefits and adverse outcomes of reducing sodium intake in the population (Institute of Medicine. 2013. *Sodium Intake in Populations: Assessment of Evidence.* Washington, DC: The National Academies Press, 2013). Likewise, reference 21 is also an opinion paper. It would be more precise to mention the original IOM report and to mention that there is a discrepancy between the inconclusive outcome of the original commissioned independent report intended to form the basis for the guidelines and the final definitive conclusion of the guidelines (reference: Dietary Guidelines for the Americans https://health.gov/dietaryguidelines/2015/guidelines/ (2015-2020).

**Response:** We have updated our references in line with the recommendation of the reviewer.

**Comment:** The purpose was to explore the effects of an educational intervention to reduce sodium intake targeting low levels. This is the right question, because the recommendations are not just to lower sodium intake, but to lower sodium intake to low levels below 2.3 g, the problem being that there are no studies to support beneficial health effects of such a reduction. Furthermore, the COSTICK study includes an outcome, which is not just a “biochemical” outcome, but a true morbidity outcome, as it reflects the effect on the renal function over a 2-year period. In contrast, the COSIP study “only” reflects cardiovascular biomarkers, but some of them have not been investigated previously and may contribute to establish whether a low sodium diet changes the disposition (for instance increases or decreases hsTnT and proBNP) to true cardiovascular events. Although it must be expected that aldosterone and renin will increase during the low sodium diet according to reference 9 and a new study (DOI: https://doi.org/10.1016/j.eclinm.2021.100750) ^1^ , it is worth to investigate this effect over a 2-year period.

**Response: **We thank the reviewer for this comment.

**Comment:** The diet counselling is more intense in the intervention group, which leaves the question whether there will be other dietary differences between the two groups than differences in sodium intake (Table 2). Such interactions may complicate the final interpretation of the results. The many 24-h urine measurements (Table 3) eliminate one of the previous issues of conflict (the method of sodium intake measurement), which has resulted in reservations on the interpretation of outcomes in previous studies.

**Response: **We thank the reviewer for this comment and agree that it is possible that additional dietary differences may occur as approaches to alter diet to reduce sodium intake may alter the intake of other nutrients, particularly potassium. As such, we are measuring 24hour urine potassium excretion throughout the trial.

**Comment:** The study is well designed, the statistical procedures are appropriately described, and the level of sodium reduction is controlled by multiple 24-h urine measurements. These preconditions should ensure solid conclusions. The authors state that “These trials will help determine the feasibility of sodium restriction to current guideline levels and determine if this reduction in intake is sustainable.” This is an important point, supported by the fact that the duration of sodium reduction in RCTs sponsored by the National Heart, Lung and Blood Institute decreased from 36 months (The Hypertension Prevention Trial: *Arch Intern Med* 1990; 150: 153–162) to 6 months (The Trials of Hypertension Prevention, *JAMA* 1992; 267: 1213–1220 and *Arch Intern Med *1997; 157: 657–667) to 1-month (the Dietary Approaches to Stop Hypertension (DASH) diet *N Engl J Med* 2001; 344: 3–10). The obvious explanation for this change in duration strategy from 36 months to 1 month is that the sodium reduction and blood pressure effects achieved in the longer-term HPT and TOHP studies were small. This leaves some questions. Why is the official policy to reduce sodium intake to below 2.3 g, if this strategy is not feasible for more than 1 month, even under controlled circumstances? And why did NHLBI support a short-term sodium reduction study, knowing that a sodium reduction strategy to below 2.3 g was not feasible for a longer period? In this context it will be exciting to learn whether the present studies will show that such a strategy is possible or not, and it will be important to interpret the outcomes in relation to the achieved sodium reduction. If the goal to reduce sodium intake to below 2.3 g is not achieved, it is reasonable to raise the question why it is difficult to get below that level. Is it because the normal human neuro-endocrinological physiology counteracts a sodium intake below 2.3 g, simply because the net-effect of such a low sodium intake is harmful, as indicated by the activation of RAAS at sodium intakes below 2.3 g? In this context it is predictable that renin and aldosterone will only increase significantly, if the goal to reduce sodium intake below 2,3 is fulfilled.

**Response: **We thank the reviewer for this comment and look forward to exploring such issues in the analysis and dissemination of our trial findings.

**Comment: **From a scientific and evidence-based point of view the outcomes of the COSTICK and COSIP studies will be exciting. If the studies show the “right” results and support the present sodium reduction policy, the studies will be well-received by the health authorities. But, if the results “wrongly” are in opposition to the existing sodium reduction policy, they will have no impact, because the political decision makers in the health organizations for decades have explained away studies, which do not confirm the official policy.

**Response: **We thank the reviewer for this comment.
